# Supplementary material for: Docking and Molecular Dynamics Predictions of Pesticide Binding to the Calyx of Bovine β-Lactoglobulin
Source: Int J Mol Sci. 2020 Mar 14;21(6):1988. doi: 10.3390/ijms21061988 (PMC7139891; doi:10.3390/ijms21061988)
Supplement: Supplementary file 1 [file ijms-21-01988-s001.pdf]

A

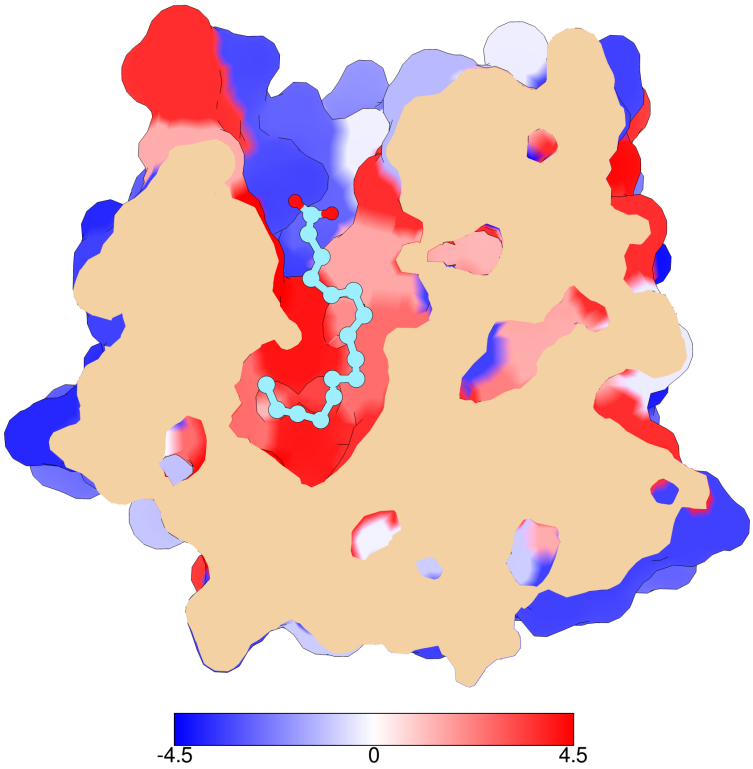

Figure S1. BLG monomer (PDB-ID 1GXA) emphasizing its binding site, or “calyx”. The palmitate shown is the docked ligand (lightblue). The colored surface indicates Kyte and Doolittle hydrophobicity with 4.5 (red)corresponding to Ile and -4.5 (blue) to Arg. More details about the binding site can be found in reference (15).
